# Undifferentiated carcinoma of the liver with osteoclast-like giant cells: a case report and literature review

**DOI:** 10.1186/s13027-024-00582-7

**Published:** 2024-04-20

**Authors:** Lixia Lu, Li Wang, Can Peng, Li Chen, Ximan He, Chenning Shao, Chunnian Wang, Rong Ge

**Affiliations:** Department of Histopathology, Ningbo Clinical Pathology Diagnosis Center, No. 685 East of North Huangcheng Road, Jiangbei District, Ningbo City, 315021 Zhejiang Province China

**Keywords:** Liver, Undifferentiated carcinoma, Osteoclast-like giant cell, Diagnosis

## Abstract

Hepatocellular carcinoma (HCC) is the most common primary malignant tumor of the liver. Osteoclast-like giant cells (OGCs) are relatively more common in pancreatic cancer, but extremely rare in HCC. Currently, there have been only a few reported cases of OGCs in HCC, and their presence indicates an aggressive clinical course. Here, we present a case of primary undifferentiated carcinoma of the liver with OGCs in a 49-year-old male patient, and through a literature review, we summarize 20 similar cases to further understand the diagnosis, treatment, and clinical course of this disease entity.

**Supplementary Information:**

The online version contains supplementary material available at 10.1186/s13027-024-00582-7.

## Introduction

Hepatocellular carcinoma (HCC) is the most common malignant tumor of the liver [1]. In recent years, its incidence has been increasing [2], with high rates of occurrence and mortality. Chronic hepatitis B virus (HBV) infection, chronic hepatitis C virus (HCV) infection, nonalcoholic fatty liver disease (NAFLD), and cirrhosis are important etiological factors for HCC. HBV accounts for the majority cases of HCC, with most infections acquired through perinatal and early horizontal transmission.

Carcinomas with osteoclast-like giant cells (OGCs) occasionally occur in a variety of sites such as pancreatic [3], ampullary [4], duodenal [5], gastric [6], gallbladder [7], thyroid [8], breast [9], lung [10], urinary bladder [11], ureter [12], kidney [13], cutaneous [14], parotid gland [15], renal pelvis [16], salivary [17], ovary [18], and liver [19], with pancreatic tumors being the most common. The presence of OGCs in HCC is extremely rare, and there have been relatively few clinical reports. Without understanding its clinicopathological characteristics, there is a risk of misdiagnosis and delayed diagnosis, resulting in poor prognosis. Since Munoz et al. first described this phenomenon in 1980 [19], only 19 similar cases have been reported [19–37], and the follow-up information for these cases suggests aggressive biological behavior. Here, we report a rare case of undifferentiated carcinoma of the liver with OGCs and review similar liver cases published between 1980 and 2023. We discuss the epidemiology, clinical presentation, pathological features, treatment, and prognosis of this disease to systematically gather more information and provide evidence for its diagnosis and treatment.

## Case preparation

**Fig. 1:**
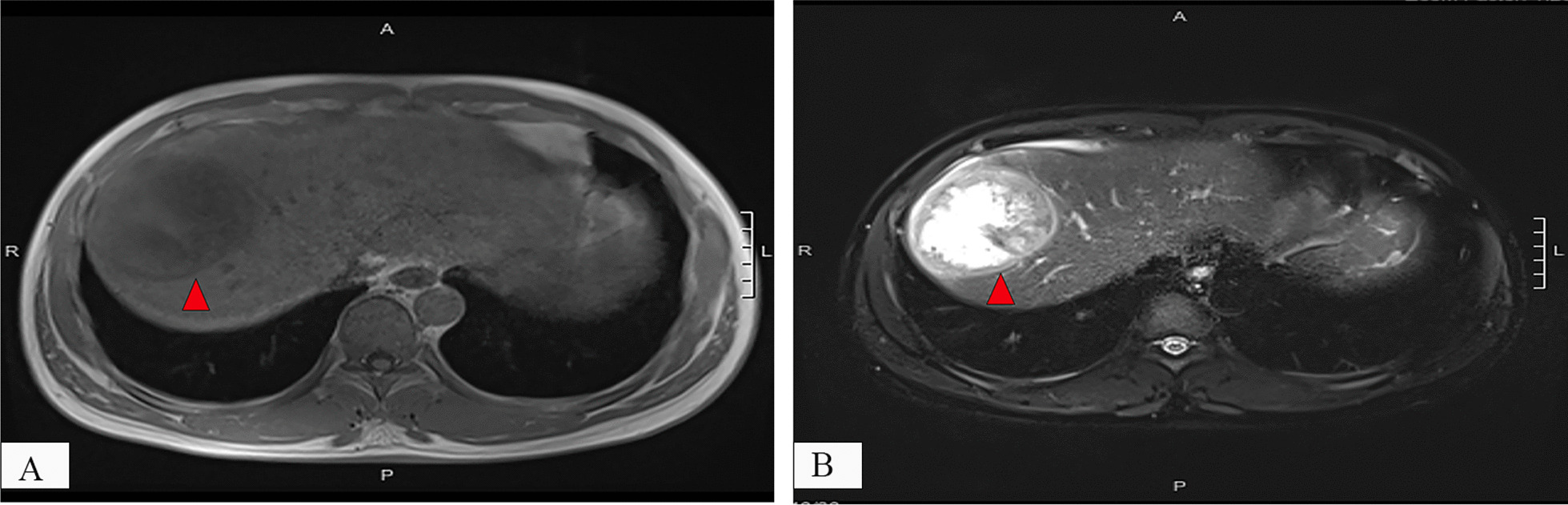
Abdominal MRI findings in HCC with OGCs. T1-weighted image showing a round-shaped hyperintense lesion (red arrow) with heterogeneous internal signal intensity (**A**). After contrast enhancement, it demonstrates a ring-enhancing pattern (red arrow) (**B**)

The patient was a 49-year-old male with over 20 years of chronic hepatitis B virus (HBV) infection. He was taking entecavir for treatment. Six months prior to admission, abdominal ultrasound suggested a liver mass suspected to be a vascular tumor, but no treatment was given. Recently, the patient complained of discomfort and pain in the right upper abdomen and was admitted to the hospital. MRI of the upper abdomen showed a circular T1/T2 signal shadow in the right lobe of the liver, measuring approximately 7.7 × 0.7 × 6.4 cm. After enhancement, it showed annular reinforcement. Multiple small round low-signal shadows without enhancement were also visible in the liver, and no obvious abnormalities were found in the gallbladder or pancreas, suggesting malignancy (Fig. 1). The tumor markers showed that alpha-fetoprotein (AFP) was 6.2 ng/ml and carbohydrate antigen (CA) 19-9 was 6.2 U/ML. The patient had no history of hypertension or diabetes. After excluding contraindications, the patient underwent surgical resection of the IVb + V + VIII segments of the liver. He was discharged from the hospital 14 days after surgery without any complications and was in good clinical condition.

## Pathological findings

**Fig. 2:**
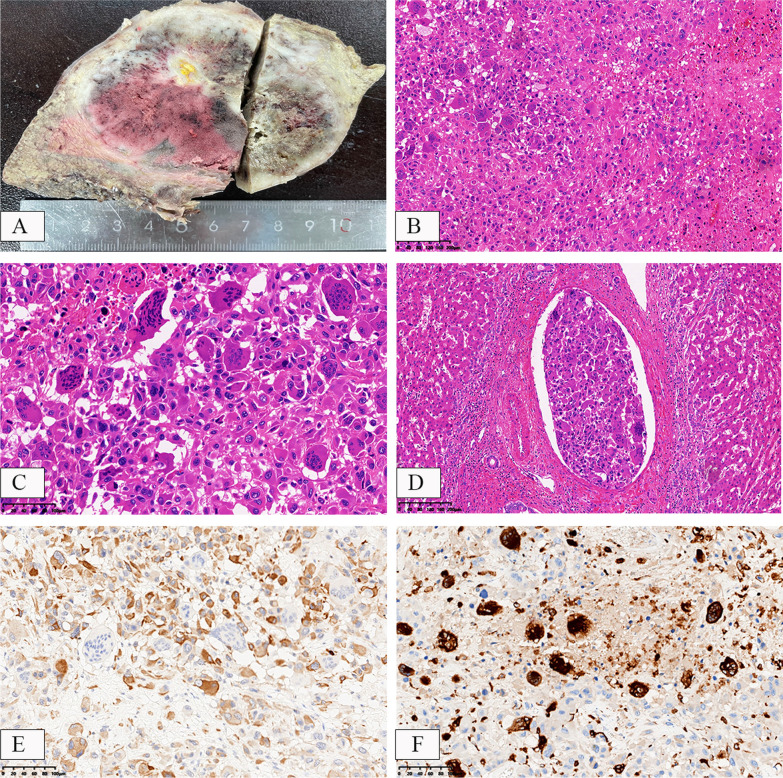
Macroscopical and histopathological findings in HCC with OGCs. Macroscopically, the tumor section appears solid with local evidence of bleeding, necrosis, and a gray-white, hard texture (**A**). The tumor is composed of two components: pleomorphic mononuclear cells and osteoclast-like giant cells (**B**, **C**) with intravascular tumor emboli (**D**). Immunohistochemical staining shows positive expression of cytokeratin (AE-1/AE-3) in mononuclear cells and negative expression in OGCs (**E**); tissue macrophage marker (CD68) is negative in the mononuclear cell area but strongly positive in the OGCs area (**F**). H&E staining: **A**, **B**, **D** × 100, **C **× 200. Immunohistochemical staining: **E**, **F** × 200

Macroscopic examination revealed an irregular solid tumor with areas of hemorrhage and necrosis (Fig. 2A). Microscopic examination revealed two components of the tumor. The first component consisted of mononuclear cells that were oval or spindle-shaped, with deeply stained nuclei, prominent nucleoli, eosinophilic cytoplasm, marked pleomorphism, frequent mitoses, and extensive necrosis and hemorrhage. The second component consisted of clusters of osteoclast-like giant cells (OGCs). There was no evidence of transition between the tumor cells and OGCs (Fig. 2B, C). Occasionally, intravascular tumor thrombi were observed in the surrounding liver tissue (MVI = M1) (Fig. 2D). The surrounding liver tissue showed cirrhotic changes. Immunohistochemistry staining showed positive expression of cytokeratin (AE-1/AE-3) in mononuclear cells (Fig. 2E), while CK8/18, CK7, CK19, and hepatocyte, arginase-1, and AFP were all negative. The tissue cell markers CD68 and vimentin were negative in mononuclear cells but strongly positive in OGCs (Fig. 2F). OGCs were negative for epithelial markers (AE-1/AE-3, Cam5.2). EBER in situ hybridization showed negative results. Other immunostains, such as SOX10, melanoma, and H3.3G34W, were all negative.

Based on the above results, the patient was diagnosed with poorly differentiated carcinoma with OGCs. The patient was followed up for 40 days after surgery and did not receive any adjuvant radiation or chemotherapy. Currently, the patient is in good condition.

## Literature review

A literature search on PubMed for hepatocellular carcinoma (HCC) with osteoclast-like giant cells (OGCs) yielded 20 cases, including our case, without the exception of one Japanese language article. Clinical and pathological information, such as gender, age, tumor size, clinical presentation, neoadjuvant therapy, underlying liver disease, location of surgery, histopathological diagnosis, cirrhosis, CD68 expression in OGCs, and clinical outcomes were collected for each patient. Detailed clinical and pathological information for these cases, including our case, is presented in Additional file 1: Table S1 [19–37].

## Discussion

Hepatocellular carcinoma (HCC) with osteoclast-like giant cells (OGCs) is extremely rare and was first described and reported by Munoz [19]. To date, including our case, there have been only 20 reported cases [19–37]. Westra et al. [24] reported a liver consultation case without detailed patient information. Tsukimoto et al. [35] reported a case of HCC recurrence with OGCs, while the remaining cases were primary liver cases. One case was a postmortem examination [28]. This study highlights the aggressive clinical course and poor prognosis of HCC with OGCs.

HCC with OGCs predominantly affects males, with 15 male patients and 4 female patients in this series (male-to-female ratio of 3.75:1). The age of onset ranged from 42 to 87 years (median age 68 years, average age 66 years). Tumor size ranged from 2.1 to 12 cm (mean size 7.43 cm). Common clinical symptoms included abdominal pain, nausea, high fever, and weight loss. Elevated blood AFP levels were observed in 4 cases. Three patients received transarterial embolisation (TAE) or transarterial chemoembolisation (TACE) prior to surgery. 11 patients had concurrent hepatitis (55%), and 11 cases showed cirrhosis in the surrounding liver tissue (55%).

HCC with OGCs is an extremely rare pathological subtype, with undifferentiated carcinoma accounting for 9.45% of cases, conventional hepatocellular carcinoma accounting for 7.35%, sarcomatoid carcinoma accounting for 3.15%, and lymphoepithelial carcinoma accounting for 1.5%. Microscopically, the tumor is composed primarily of two components: a population of markedly atypical mononuclear cells and OGCs. The mononuclear cells express epithelial or hepatocellular markers, while OGCs express tissue histiocyte markers (CD68) but do not express epithelial markers.

There is considerable controversy regarding whether OGCs constitute a separate tumor entity. Westra [24] analyzed K-ras gene mutations in five cases of pancreatic and hepatic tumors containing OGCs and found that four cases had identical point mutations in both the mononuclear tumor cells and OGCs. Furthermore, the two components showed similarities in ultrastructure, suggesting that OGCs may arise from fusion between infiltrating mononuclear tumor cells. However, some researchers believe that the tumor entity consists only of mononuclear cells, while OGCs are formed by recruitment and fusion of mononuclear tissue histiocytes/macrophages derived from the bone marrow in response to chemotactic factors produced by tumor cells [4, 38–40]. Rosai [41] proposed that multinucleated giant cells originate from non-epithelial cells with osteoclast phenotypes and are fundamentally non-neoplastic. Sakai [42], using microdissection analysis, investigated the origin of giant cells in three cases of pancreatic cancer with OGCs. In each case, no K-ras gene mutations were detected in microdissected OGCs, but tissue histiocyte marker (CD68) expression was positive. K-ras gene mutations were detected in the ductal carcinoma cells. Therefore, it is believed that OGCs have a different origin from ductal carcinoma cells and are strongly suggested to be non-neoplastic and of mesenchymal origin. Immunohistochemical staining of our case showed loss of p53 protein expression in the mononuclear cell population, indicating TP53 gene mutation, while wild-type expression was observed in the OGC area. Ki-67 expression was approximately 20% in the mononuclear cell population, while it was almost absent in the OGC area. CD68 and Vimentin showed strong positive expression in OGCs. From an immunohistochemical perspective, our study suggests that the tumor entity consists only of mononuclear cells, while OGCs are non-neoplastic and non-epithelial, which is consistent with the findings of Sasaki [23].

Differential diagnosis includes several subtypes of HCC that may show multinucleated cells. Hepatocellular carcinoma with syncytial giant cells is a special variety of liver tumor, described in both paediatric and adult populations. The multinucleated giant cells in the present HCC were clearly epithelial and probably hepatocyte in origin based on the distinctive immunophenotype with reactivity for a hepatocyte marker and cytokeratin 8 [43]. Sarcomatoid HCC may show areas of mesenchymal differentiation with multinucleated giant cells [25], featured by reactivity for CK 8, ALB, and fibrinogen, as well as for Vimentin [44]. Finally, HCC with OGCs should be considered as well. The tumor in our case showed coexistence of undifferentiated carcinoma of the liver and osteoclast-like giant cells, exhibiting negativity for hepatocellular and epithelial markers and only positivity for CK-pan.

Surgical resection remains the main treatment option for HCC with OGCs. Tsukimoto [20] reported a case of HCC with OGCs that recurred 9 years after surgery. The patient underwent complete resection of the affected liver segment and radiofrequency ablation under ultrasound guidance. The patient remained recurrence-free for one and a half years after the surgery, which is the longest reported survival period to date.

HCC with OGCs has a poor prognosis [26]. Macrophages, as immune effector cells, have been proposed two distinct states of polarized activation for macrophages: the classically activated (M1) macrophage and the alternatively activated (M2) macrophage subsets [45]. M2 macrophages in cancer stroma has been considered to be an important factor in the acceleration of malignant behavior in cancers. They express a series of cytokines, chemokines, tumor growth, metastasis, and immunosuppression [46]. Sajjadi [47] found several similarities between OGCs and M2 tumor-associated macrophages, particularly in their morphology and immunophenotype, and a miRNA monocytic signature. Hatano [48] established an in vivo OGC maturation model, and OGCs in the tumor environment accelerated the growth of tumors independent of macrophage colony-stimulating factor or receptor activator of nuclear factor-kappa B ligand. They revealed that OGCs in the tumor environment promoted tumor growth and lymphangiogenesis by secreting vascular endothelial growth factor-C. Taken together, these findings indicate that OGCs can promote tumor angiogenesis, growth, and metastasis. Clinically this type of tumor is very aggressive. So, mediating macrophage to resist tumors may provide more efficacious novel therapies for future tumor management. In a literature review of 19 cases, 2 cases experienced recurrence, 11 cases died within 4 months after surgery, and only 1 case had a favorable outcome. In this case, there were occasional intravascular tumor emboli (MVI = M1), and the surrounding liver tissue showed evidence of cirrhosis, but no distant metastasis was observed. The patient's current condition is stable, but close follow-up is required in the nearly future.

## Conclusion

In conclusion, HCC with OGCs is a very rare condition with an aggressive clinical course, suggesting a poor prognosis. Mediating macrophage to resist tumors may provide more efficacious novel therapies for future tumor management. Due to limited reported cases, further large-scale research is needed to better understand the clinical course of this condition and improve management strategies for patients.

### Supplementary Information

**Additional file 1: Table S1.** Overview of patient characteristics in included studies. A review of the English-language literature published that reported cases of HCC with OGCs. Clinical and pathological information, such as gender, age, tumor size, clinical presentation, neoadjuvant therapy, underlying liver disease, location of surgery, histopathological diagnosis, cirrhosis, CD68 expression in OGCs, and clinical outcomes A total of 20 cases of HCC with OGCs were included.

## Data Availability

The original contributions presented in the study are included in the article/ supplementary material, further inquiries can be directed to the corresponding author.

## Abbreviations

HCC: Hepatocellular carcinoma
OGCs: Osteoclast-like giant cells
HBV: Hepatitis B virus
HCV: Hepatitis C virus
NAFLD: Nonalcoholic fatty liver disease
AFP: Alpha-fetoprotein
CA: Carbohydrate antigen
